# Changes in community assembly may shift the relationship between biodiversity and ecosystem function

**DOI:** 10.3389/fmicb.2014.00424

**Published:** 2014-08-13

**Authors:** Joseph E. Knelman, Diana R. Nemergut

**Affiliations:** ^1^Department of Ecology and Evolutionary Biology, University of ColoradoBoulder, CO, USA; ^2^Institute for Arctic and Alpine Research, University of ColoradoBoulder, CO, USA; ^3^Environmental Studies Program, University of ColoradoBoulder, CO, USA; ^4^Department of Biology, Duke UniversityDurham, NC, USA

**Keywords:** biodiversity-ecosystem functioning, community assembly theory, niche vs. neutral, stochastic vs. deterministic, rare organisms, biodiversity dilution effect

Can differences in community assembly alter the relationship between biodiversity and ecosystem function? Pholchan et al. (2013) used a variety of manipulations to change microbial community assembly in sludge reactors and examined the subsequent links between diversity and a rare function, the removal of endocrine disrupting compounds (EDCs). Interestingly, the authors saw no consistent differences between shifts in alpha diversity (e.g., species richness and evenness) and ecosystem function, observing an increase, decrease and no difference in the amount of removal of specific EDCs with increases in diversity. They suggested that differences in community assembly may be driving variation in the relationship between biodiversity and function, a fascinating hypothesis that unites processes in community and ecosystem ecology.

Combinations of four processes affect community assembly: dispersal and diversification add new taxa to communities while selection and drift affect their relative abundances (Vellend, 2010; Nemergut et al., 2013). Particular research emphasis has been placed on assembly processes that are driven by differences between taxa (“niche”) compared to those in which any such differences are irrelevant to fitness (“neutral”) (Hubbell, 2001). Likewise, researchers have focused on the role of stochasticity, where assembly is more probabilistic vs. determinism, in which randomness does not affect community dynamics. Niche and neutral processes can operate in unison (Adler et al., 2007) and both can be affected by stochastic and deterministic forces (Fox, 2012). Indeed, extensive data demonstrate that a variety of factors, including nutrients, productivity, resource availability, successional stage, and disturbances may affect the relative importance of different community assembly mechanisms (Chase, 2007, 2010; Ferrenberg et al., 2013; Kardol et al., 2013). However, to our knowledge, no studies have directly tested how shifts in community assembly may affect the relationship between biodiversity and ecosystem function.

Of course, a great deal of research has focused on pairwise combinations of the interactions between community assembly, biodiversity and/or function in isolation. First, a large body of work demonstrates links between biodiversity and ecosystem function (Cardinale et al., 2011; Hooper et al., 2012), even for microbial systems (Bell et al., 2005; Hsu and Buckley, 2009; Langenheder et al., 2010; Levine et al., 2011; Jousset et al., 2014). However, the nature and strength of biodiversity ecosystem function (BEF) relationships have been widely debated and strongly depend on the type of function and ecosystem examined (Grime, 1997; Hooper et al., 2005) and the degree of redundancy within the community (Reich et al., 2012; Jousset et al., 2014). These complexities may be heightened for microorganisms due to the extraordinary phylogenetic diversity harbored within microbial communities, and the fact that a typical microbial community contains organisms from within a variety of functional guilds.

Second, it is known that different assembly mechanisms drive biodiversity in distinct ways. For example, spatial or temporal variation in environmental conditions increases biodiversity through niche processes while increases in the diversity of the metacommunity or in the ratio of immigration/emigration rates can increase biodiversity through neutral processes (Vellend, 2010).

Finally, a relatively new topic in the literature relates community assembly and ecosystem function (Fukami et al., 2010; Nemergut et al., 2013). Vital to such a consideration is the relationship between response traits, or traits that can interact with environmental variation to determine species distribution and abundance patterns, and effect traits, or traits that determine the functional roles of different taxa (Naeem and Wright, 2003). When communities are largely structured by niche processes, variation in the environment can directly correlate to effect traits that are linked to selected response traits (Allison, 2012). However, when communities are structured by neutral processes, ecosystem function will primarily depend on effect trait abundances within the metacommunity, dispersal and ecological drift; thus, relationships between variation in the environment and effect traits can be decoupled (Nemergut et al., 2013). Communities can also be structured by niche-based processes that act on response traits that are unrelated to the effect trait, i.e., the ecosystem process of interest, again resulting in a lack of a relationship between the environment and effect traits (Jiang et al., 2008).

Thus, various studies have examined separate pieces of the assembly-BEF puzzle, but we know of no work that explicitly ties all three factors together. However, given that niche and neutral processes partially underpin the proposed mechanisms driving positive BEF relationships (Loreau and Hector, 2001), examining the links between assembly, biodiversity and ecosystem function simultaneously, rather than in pairwise combinations, may yield new insights into the controls and consequences of biodiversity. For example, niche complementarity occurs when a more diverse community occupies a greater diversity of niches and thus can have greater overall functional efficiency (Figure 1A). Alternatively, neutral processes can lead to increases in diversity through increases in immigration, a phenomenon that may be particularly important in early succession (Ferrenberg et al., 2013). This could lead to a sampling effect in which more diverse communities include members with an effect trait of interest as the community equilibrates with the metacommunity, thus having a higher rate of function per capita (Figure 1B). Thus, because different assembly processes can lead to communities with different community compositions but with the same level of alpha diversity, assembly may lead to differences in nature of BEF relationships.

**Figure 1:**
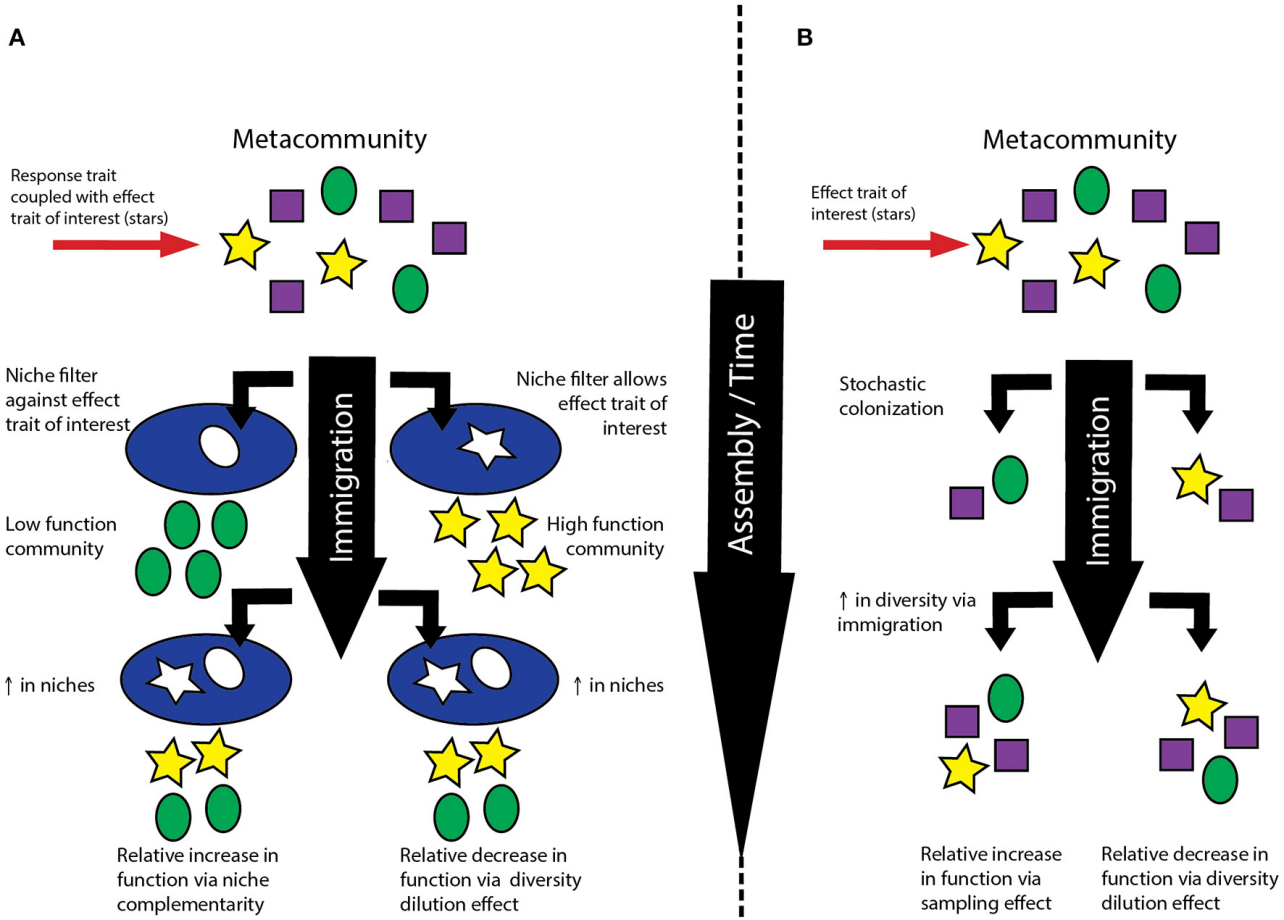
**(A)** Relationship between niche-driven increases in biodiversity and ecosystem function. Shapes represent traits where response and effect traits are directly coupled; the trait of interest is represented as a yellow star. Depending on the initial community, increases in diversity can lead to increases in relative function through niche complementarity or decreases through the biodiversity dilution effect. **(B)** Relationship between stochastic, neutrally-driven increases in biodiversity and ecosystem function. The trait of interest is represented as a yellow star. Increases in diversity can lead to increases in relative function through a sampling effect if the trait of interest is absent from the initial community. However, increases in diversity could also result in decreases in relative function through the biodiversity dilution effect if the trait is present in the initial community. The biodiversity dilution effect may be especially important if the trait is more rare in the metacommunity but stochastic processes result in its appearance in the initial community.

Additionally, depending on the starting conditions of the community and the degree of stochasticity, it is also possible that different assembly processes could lead to declines in ecosystem function coincident with increases in biodiversity through changes in the “mass ratio” (Grime, 1998). In early successional communities that contain the effect trait of interest, increases in diversity driven by both niche and neutral process could lead to decreases in the amount of ecosystem function per unit biomass (Figures 1A,B). When stochastic immigration processes result in early communities with an effect trait of interest that is present in lower abundance in the metacommunity, increases in diversity driven by immigration could lead to decreases in ecosystem function per unit biomass (Figure 1B). The inverse of this relationship was reflected in the neutral model generated by Pholchan and coworkers, which showed that decreases in diversity could lead to increases in the relative abundance of rare taxa. Likewise, if a coupled response-effect trait of interest is present in an early successional community, niche-driven increases in diversity could also lead to decreases in function per unit biomass (Figure 1A). We refer to both of these scenarios as examples of *biodiversity dilution effects*, conceptually akin to the disease dilution effect (Keesing et al., 2006). Indeed, rare ecosystem functions such as EDC removal may be catalyzed by a very select group of organisms, and thus the activity of interest could be related to the presence or absence of specific taxa and particularly sensitive to diversity dilution effects.

Thus, biodiversity dilution effects, differences in response and effect traits, niche complementarity and sampling effects may have interacted in poorly understood ways to produce the lack of a consistent relationship between biodiversity and EDC degradation across the different treatments in the work presented by Pholchan and coworkers. It is important to bear in mind, however, that microbial communities are highly complex and that other factors besides assembly could affect the nature of the BEF relationship. For example, the unique resource requirements of EDC removers may have contributed to the complex relationships between diversity and ecosystem function observed in this study: Chesson (2000) showed that when organisms require highly specialized niches, they can exhibit negative frequency dependence and be more competitive in low abundance. In the research presented by Pholchan and coworkers, increases in diversity were correlated with increases in evenness, which could have affected the competitiveness and thus the overall function of EDC removers. Additionally, some ecosystem processes may be catalyzed by a consortium of organisms acting sequentially and across trophic scales; thus, ecosystem function may not be related to alpha diversity *per se*, but rather to overall community composition. The importance of species-specific traits vs. biodiversity for function has been a subject of debate for decades and appears to depend on the system and function of interest (Grime, 1997). Given the high functional and phylogenetic diversity of microbial communities and our ability to perform comparative metagenomics on a large number of samples, this should be a research priority into the future. As well, the general hypothesis put forward by Pholchan and coworkers connecting assembly processes, biodiversity and ecosystem function should be examined with directed experiments and simulations to better understand the mechanistic details of such links and when and where they may vary.

## Conflict of interest statement

The authors declare that the research was conducted in the absence of any commercial or financial relationships that could be construed as a potential conflict of interest.
